# Supplementary material for: An Evaluation of the Anticancer Properties of SYA014, a Homopiperazine-Oxime Analog of Haloperidol in Triple Negative Breast Cancer Cells
Source: Cancers (Basel). 2022 Dec 8;14(24):6047. doi: 10.3390/cancers14246047 (PMC9776707; doi:10.3390/cancers14246047)

SUPPORTING INFORMATION

An Evaluation of the Anticancer Properties of SYA014, a Homopiperazine-Oxime Analog of Haloperidol in Triple Negative Breast Cancer Cells

Gladys M. Asong[1,†], Chandrashekhar Voshavar [1,†], Felix Amissah[2], Barbara Bricker[1], Nazarius S. Lamango[1], Seth Y. Ablordeppey [1,*]

[1] College of Pharmacy and Pharmaceutical Sciences, Institute of Public Health, Florida A&M University, Tallahassee, Florida 32307, United States;
[2] College of Pharmacy, Ferris State University, Big Rapids, Michigan 49307, United States;

[*] Correspondence: seth.ablordeppey@famu.edu; Tel.: +1-850-599-3834

[†] These authors contributed equally to this work.

To whom correspondence should be addressed:
Seth Y Ablordeppey, PhD
Eminent Scholar Chair in Biomedical Sciences
Professor and Fulbright Scholar
Basic Pharmaceutical Sciences
Florida A&M University
College of Pharmacy and Pharmaceutical Sciences-Institute of Public Health
1415 S. Martin Luther King Blvd, Tallahassee, FL 32307.
Ph: 850-599-3834
Email: seth.ablordeppey@famu.edu

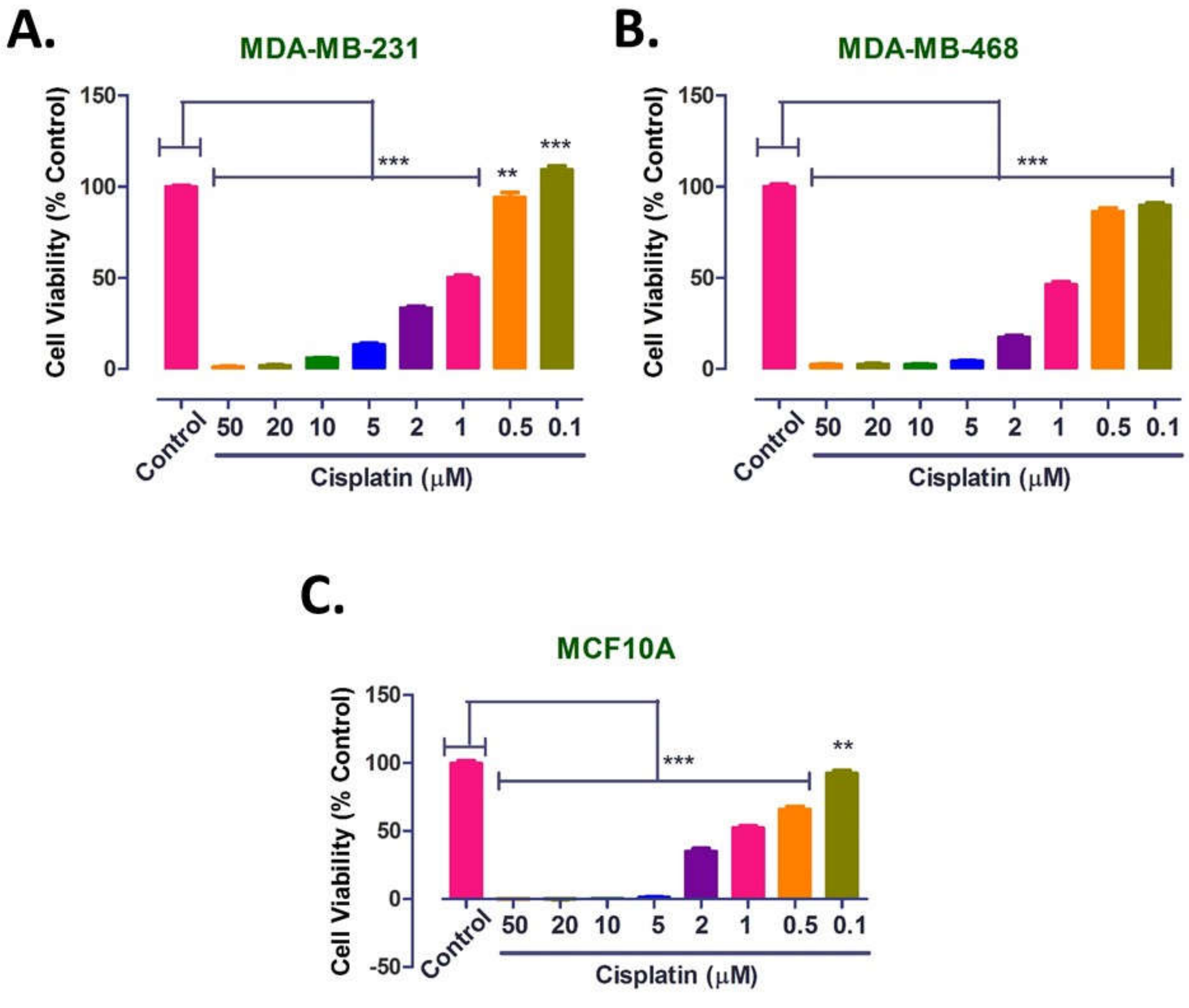

Figure S1. Cytotoxicity of Cisplatin in MDA-MB-231 (**A**), MDA-MB-468 (**B**), and MCF10A cells (**C**). Cells were treated continuously with Cisplatin for 72h. The cell viabilities were determined using resazurin reduction assay as described in the materials and methods section. Each experiment was performed in triplicate and the data points represents the mean ± SEM of 6-8 wells for each experiment. The results were plotted using GraphPad. $^{**}p < 0.01$ and $^{***}p < 0.001$.

Figure S2. Original western blot images for SYA014 in Figure 9 - A. Pro-Apoptotic Proteins**.**

Caspase 3

MDA-MB-231

SYA014 (µM)

0 2 5 10

Last 4 blots (right side) are not part of this work

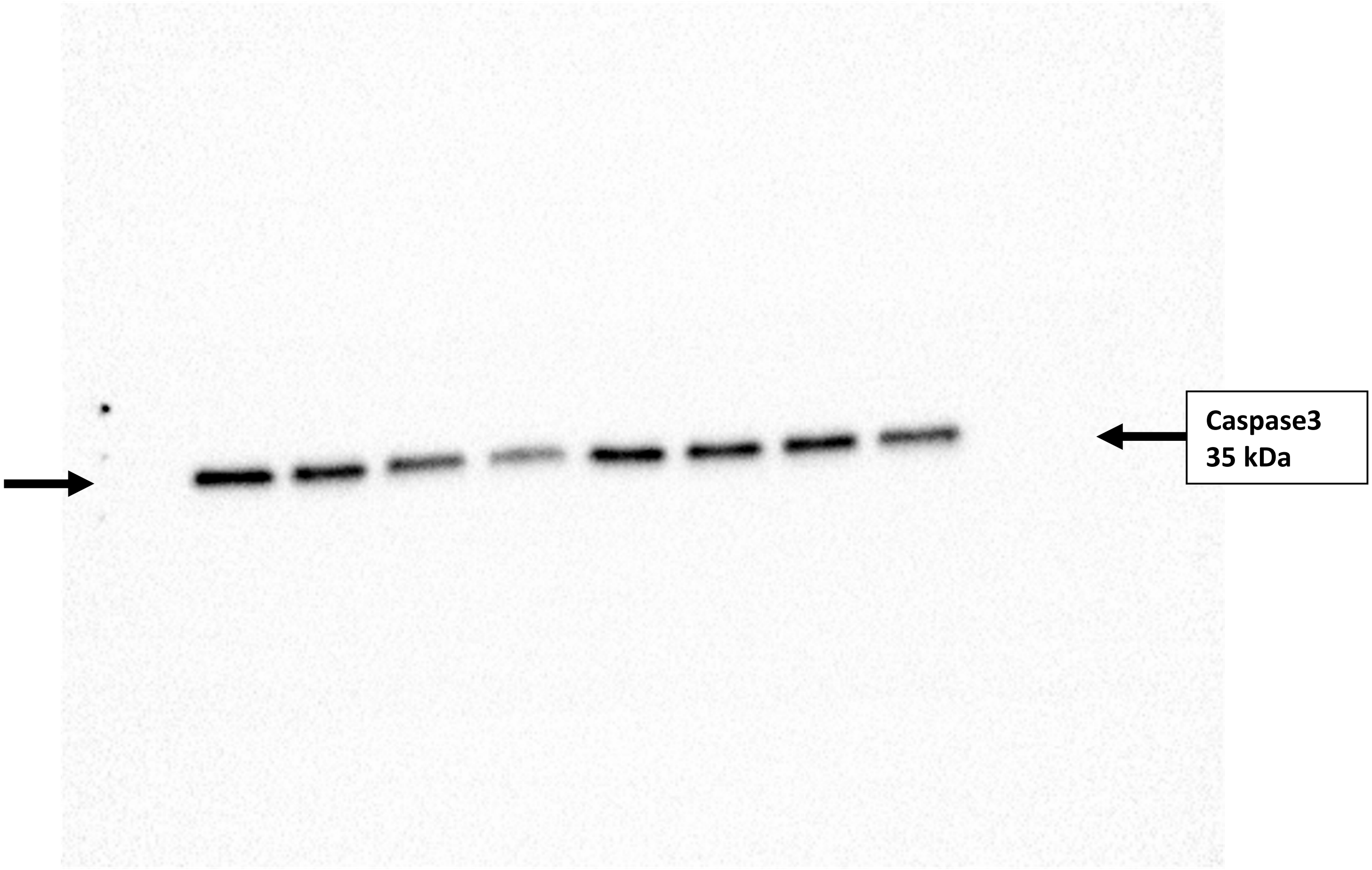

PARP

MDA-MB-231

SYA014 (µM)

0 2 5 10

Last 4 blots (right side) are not part

of this work

Image Report: Pinaki 2018-02-21 14hr 15min_Exposure_62.5sec

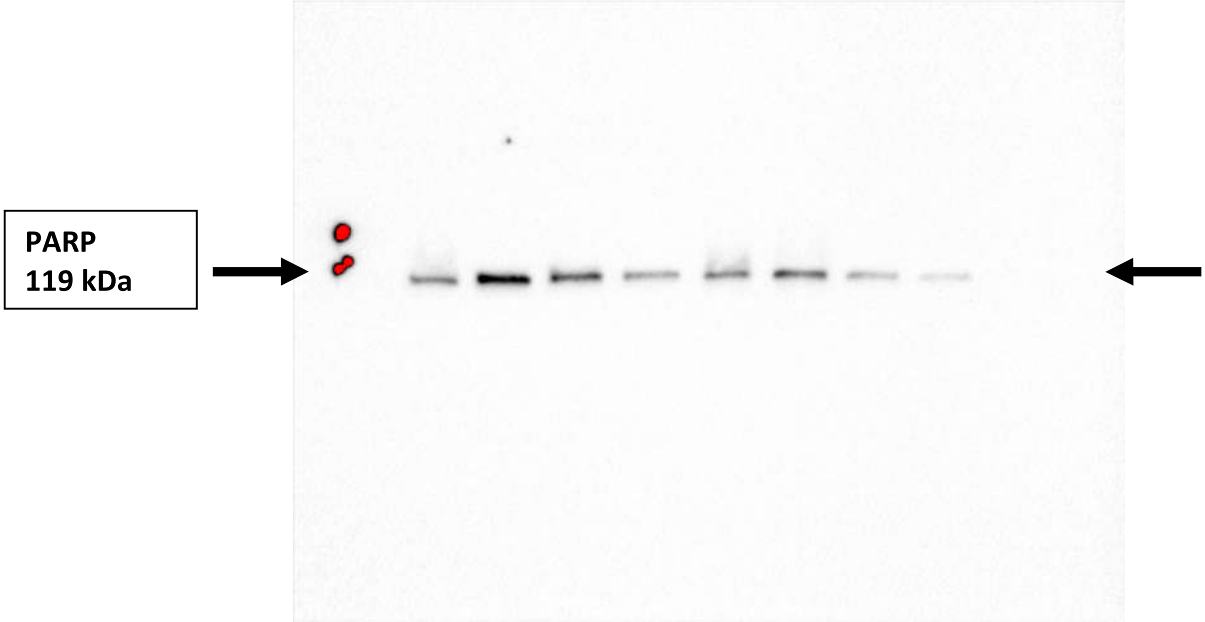

/Volumes/Seagate/Graduate Program/Research/SPECIFIC AIM 3/Western blotting/WB/Western blotting copy_2-5-18/Chemi Images/GA-2-12-18/PARP/Pinaki 2018-02-21 14hr 15min_Exposure_62.5sec.scn

Acquisition Information

Imager	ChemiDoc™ XRS+
Exposure Time (sec)	62.545 (Signal Accumulation)
Flat Field	Applied (Lens)
Serial Number	721BR12059
Software Version	5.2.1
Application	Chemi
Excitation Source	No Illumination
Emission Filter	No Filter
Binning	3x3

1

Bax

MDA-MB-231

SYA014 (μM)

0 2 5 10

Last 4 blots (right side) are not part of this work

Image Report: Pinaki 2018-03-01 14hr 22min_Exposure_2.2sec

Bax
20 kDa

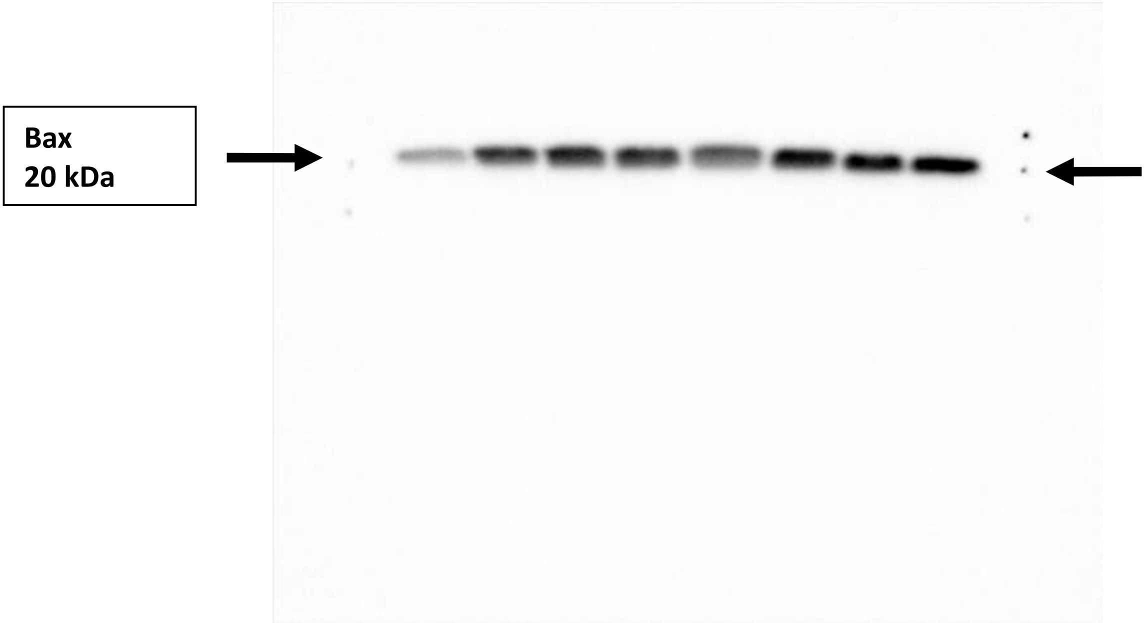

/Volumes/Seagate/Graduate Program/Research/SPECIFIC AIM 3/Western blotting/WB/Western blotting copy_2-5-18/Chemi Images/GA-2-20-18/Bax/Pinaki 2018-03-01 14hr 22min_Exposure_2.2sec.scn

Acquisition Information

Imager	ChemiDoc™ XRS+
Exposure Time (sec)	2.208 (Signal Accumulation)
Flat Field	Applied (Lens)
Serial Number	721BR12059
Software Version	5.2.1
Application	Chemi
Excitation Source	No Illumination
Emission Filter	No Filter
Binning	3x3

1

Bak

MDA-MB-231

SYA014 (μM)

0	2	5	10

Last 4 blots (right side) are not part of this work

Image Report: Pinaki 2018-03-02 12hr 20min_Exposure_1.0sec

Bak
25 kDa

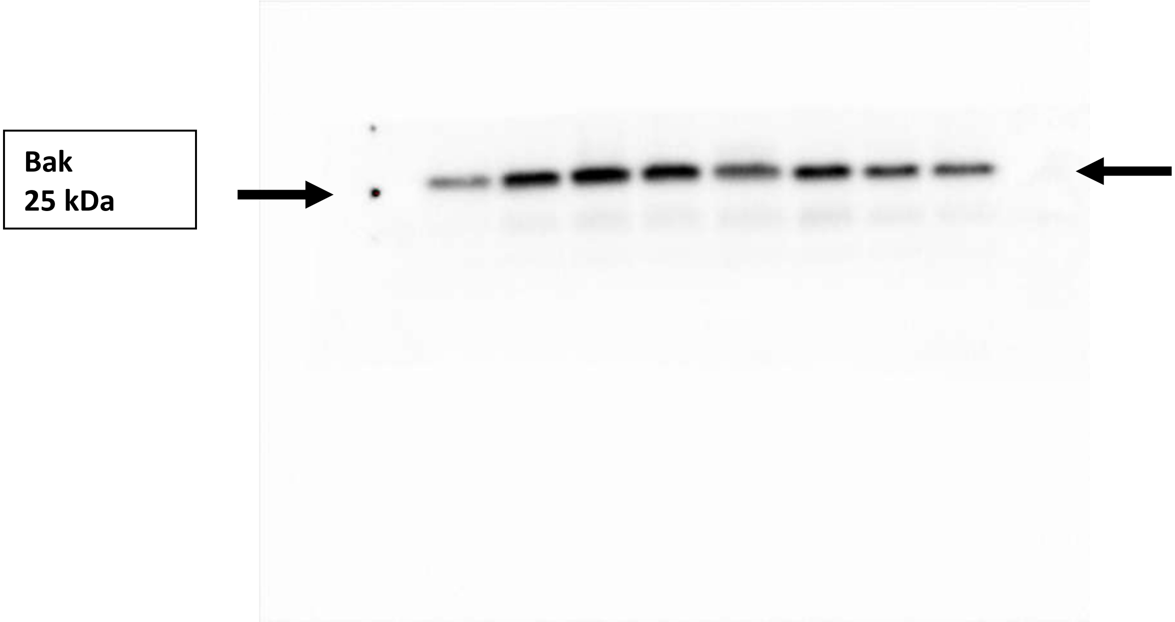

/Volumes/Seagate/Graduate Program/Research/SPECIFIC AIM 3/Western blotting/WB/Western blotting copy_2-5-18/Chemi Images/GA-2-20-18/GA-2-21-18/Bak/Pinaki 2018-03-02 12hr 20min_Exposure_1.0sec.scn

Acquisition Information

Imager	ChemiDoc™ XRS+
Exposure Time (sec)	1.000 (Signal Accumulation)
Flat Field	Applied (Lens)
Serial Number	721BR12059
Software Version	5.2.1
Application	Chemi
Excitation Source	No Illumination
Emission Filter	No Filter
Binning	3x3

1

Cytochrome C

MDA-MB-231

SYA014 (μM)

0	2	5	10

Last 4 blots (right side) are not part of this work

Image Report: Pinaki 2017-02-08 14hr 16min_Exposure_23.8sec

Cyt C
30 kDa

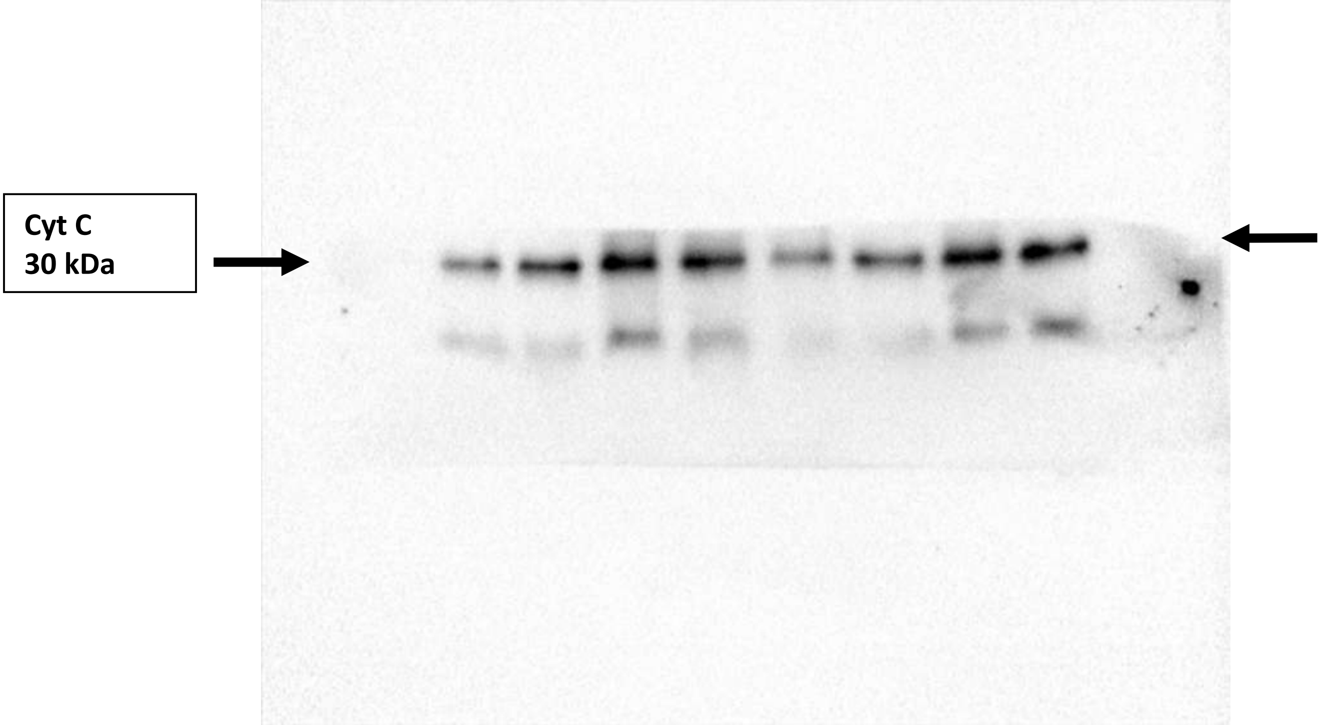

/Volumes/Seagate/Graduate Program/Research/SPECIFIC AIM 3/Western blotting/WB/Western blotting copy_2-5-18/Chemi Images/10-1-18_GA/Cytochrome c/Pinaki 2017-02-08 14hr 16min_Exposure_23.8sec.scn

Acquisition Information

Imager	ChemiDoc™ XRS+
Exposure Time (sec)	23.755 (Signal Accumulation)
Flat Field	Applied (Lens)
Serial Number	721BR12059
Software Version	5.2.1
Application	Chemi
Excitation Source	No Illumination
Emission Filt[illegible]	[illegible]
Binning	[illegible]

β-Actin

MDA-MB-231

SYA014 (μM)

0	2	5	10

Last 5 blots (right side) are r[illegible] of this work

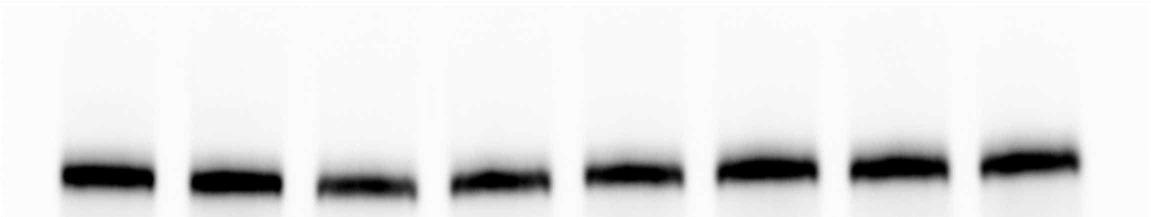

β-Actin
42 kDa

Figure 9 - C. Anti-Apoptotic Bcl-family Proteins

Bcl-2

MDA-MB-231

SYA014 (μM)

0	2	5	10

Last 4 blots (right side) are not part of this work

Image Report: Pinaki 2018-03-17 14hr 12min_Exposure_16.5sec

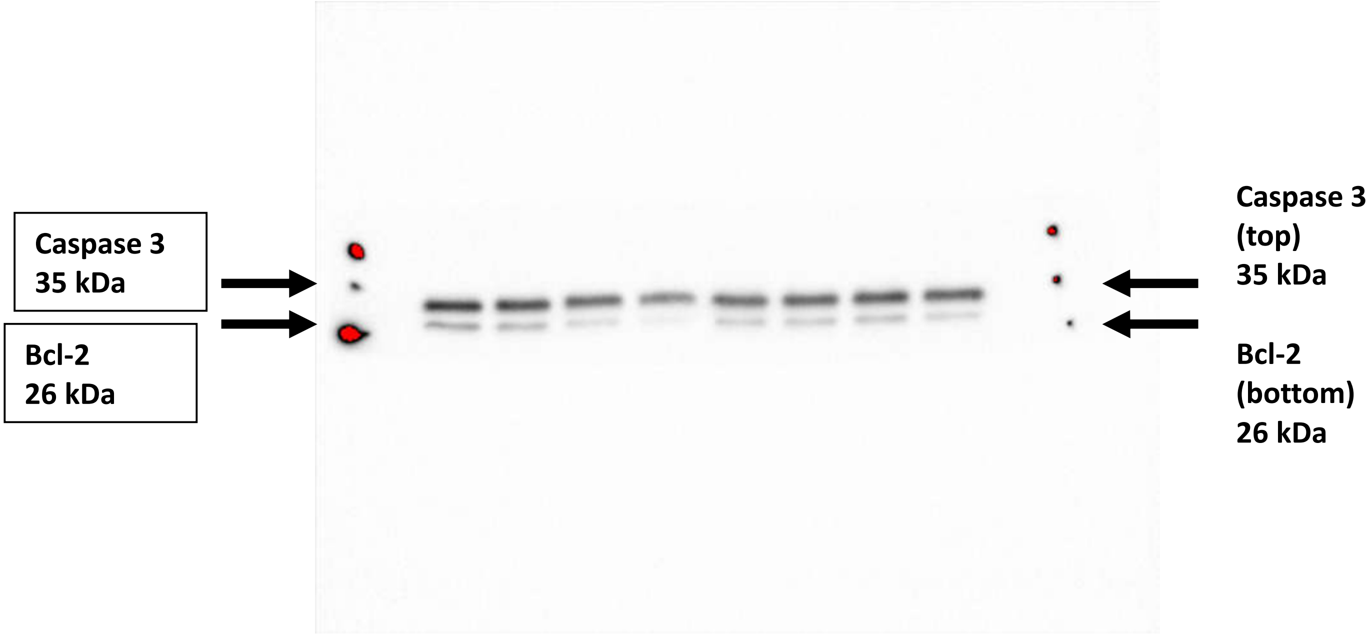

/Volumes/Seagate/Graduate Program/Research/SPECIFIC AIM 3/Western blotting/WB/Western blotting copy_2-5-18/Chemi Images/GA_3-7-18/GA_3-8-18/Bcl-2 after Casp 3/Pinaki 2018-03-17 14hr 12min_Exposure_16.5sec.scn

Acquisition Information

Imager	ChemiDoc™ XRS+
Exposure Time (sec)	16.525 (Signal Accumulation)
Flat Field	Applied (Lens)
Serial Number	721BR12059
Software Version	5.2.1
Application	Chemi
Excitation Source	No Illumination
Emission Filter	No Filter
Binning	3x3

BcL-XL

MDA-MB-231

SYA014 (µM)

0 2 5 10

Last 4 blots (right side) are not part of this work

Image Report: Pinaki 2018-03-16 12hr 40min_Exposure_105.8sec

Bcl-XL
26 kDa

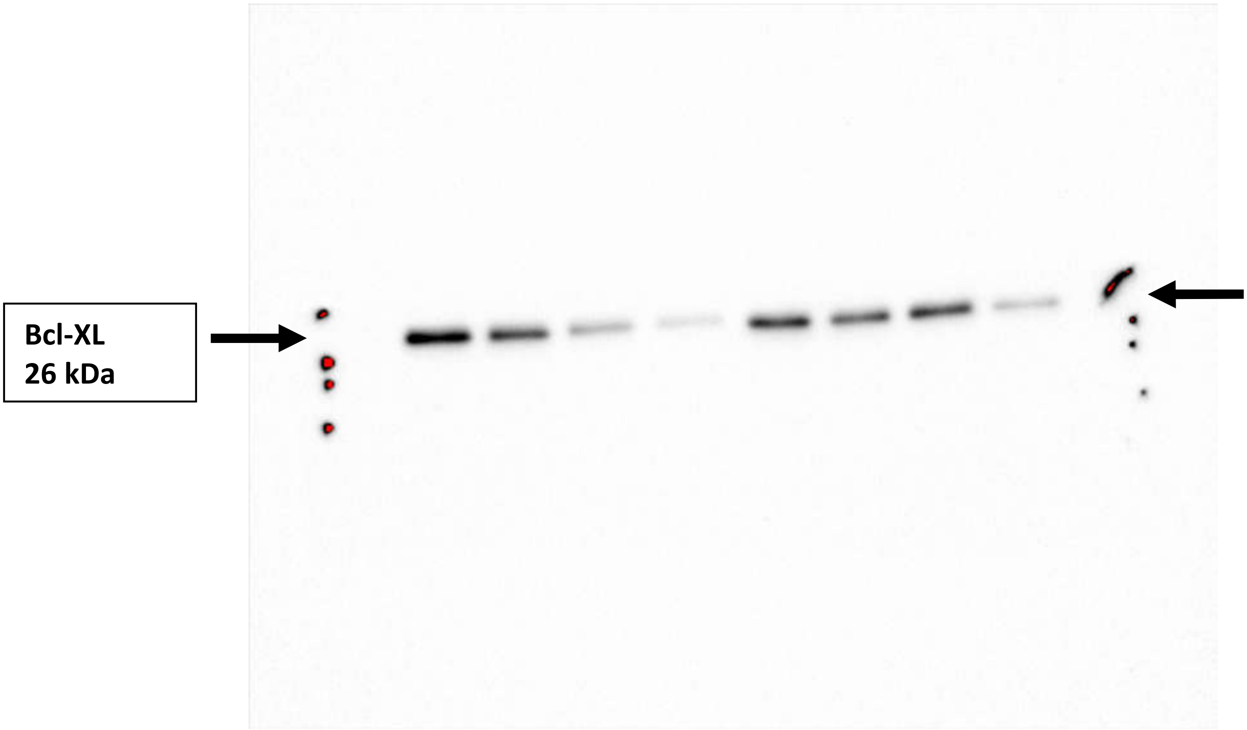

/Volumes/Seagate/Graduate Program/Research/SPECIFIC AIM 3/Western blotting/WB/Western blotting copy_2-5-18/Chemi Images/GA_3-7-18/Bcl-XL/Pinaki 2018-03-16 12hr 40min_Exposure_105.8sec.scn

Acquisition Information

Imager	ChemiDoc™ XRS+
Exposure Time (sec)	105.785 (Signal Accumulation)
Flat Field	Applied (Lens)
Serial Number	721BR12059
Software Version	5.2.1
Application	Chemi
Excitation Source	No Illumination
Emission Filter	No Filter
Binning	3x3

Mcl-1

MDA-MB-231

SYA014 (μM)

0 2 5 10

Last 4 blots (right side) are not part of this work

Image Report: Pinaki 2018-02-01 16hr 12min_Exposure_52.2sec

Mcl-1
40 kDa

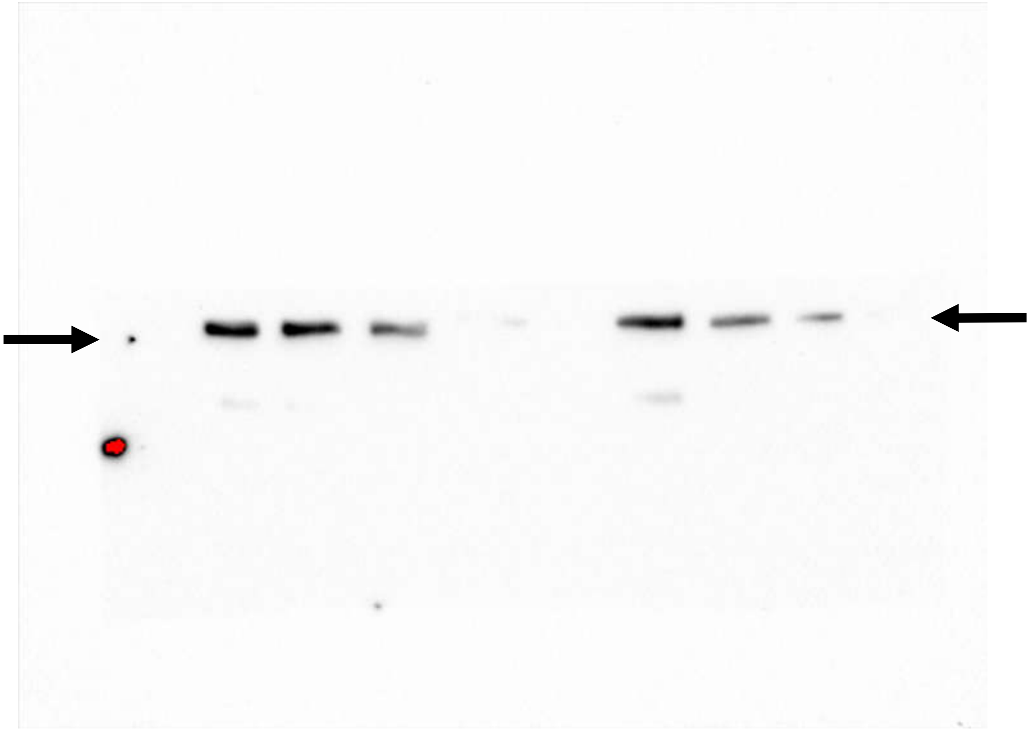

/Volumes/Seagate/Graduate Program/Research/SPECIFIC AIM 3/Western blotting/WB/Western blotting copy_2-5-18/Chemi Images/GA-1-23-18/Mcl-1/Pinaki 2018-02-01 16hr 12min_Exposure_52.2sec.scn

Acquisition Information

Imager	ChemiDoc™ XRS+
Exposure Time (sec)	52.230 (Signal Accumulation)
Flat Field	Applied (Lens)
Serial Number	721BR12059
Software Version	5.2.1
Application	Chemi
Excitation Source	No Illumination
Emission Filter	No Filter
Binning	3x3

β-Actin

MDA-MB-231

SYA014 (μM)

0 2 5 10

Last 4 blots (right side) are not part of this work

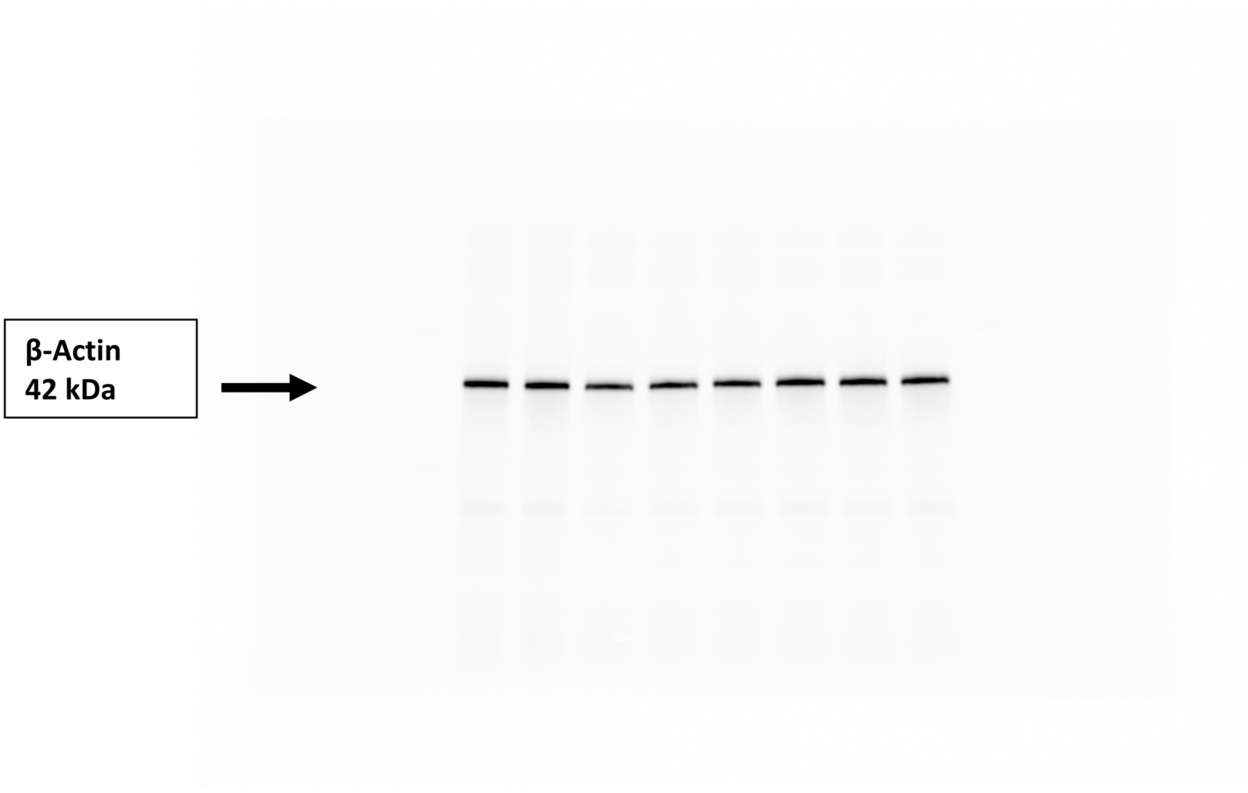

Figure 9 - E. Death Receptors
DR5

MDA-MB-231

SYA014 (µM)

0 2 5 10

Last 4 blots (right side) are not part

of this work

DR5
40 kDa
48 kDa

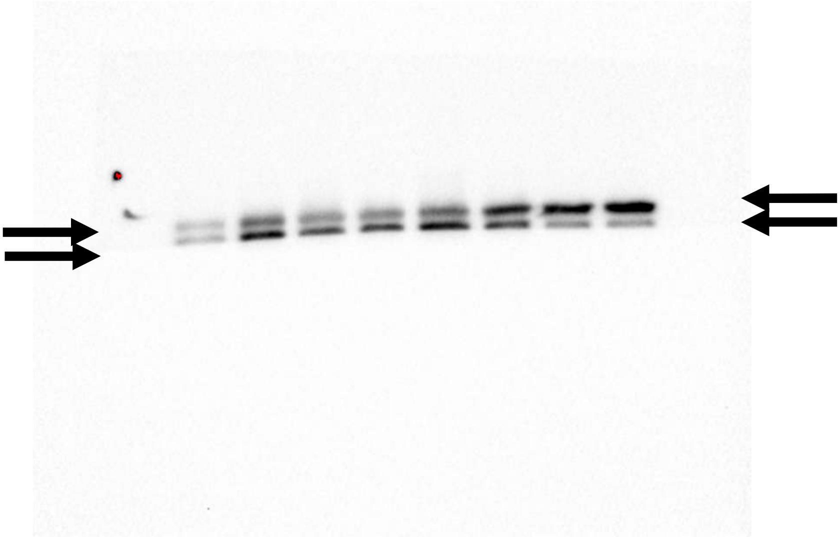

TNF-RI

MDA-MB-231

SYA014 (µM)

0 2 5 10

Last 4 blots (right side) are not part of this work

Image Report: Pinaki 2018-03-01 14hr 41min_Exposure_8.4sec

TNF-R1
55 kDa

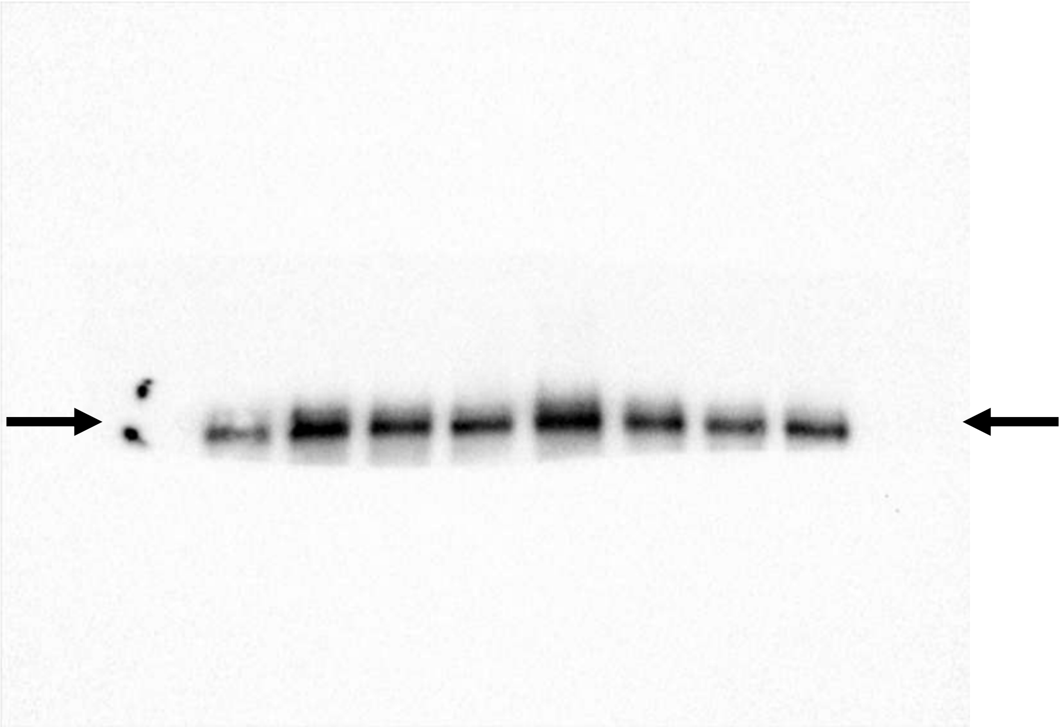

/Volumes/Seagate/Graduate Program/Research/SPECIFIC AIM 3/Western blotting/WB/Western blotting copy_2-5-18/Chemi Images/GA-2-20-18/TNF-R1/Pinaki 2018-03-01 14hr 41min_Exposure_8.4sec.scn

Acquisition Information

Imager	ChemiDoc™ XRS+
Exposure Time (sec)	8.416 (Signal Accumulation)
Flat Field	Applied (Lens)
Serial Number	721BR12059
Software Version	5.2.1
Application	Chemi
Excitation Sou	
Emission Filte	
B	

RIP

MDA-MB-231

SYA014 (µM)

0	2	5	10

First blot (left side) and last 4 blots (right side) are not part of this work

Image Report: Selected_Exposure_4.1sec

RIP
78 kDa

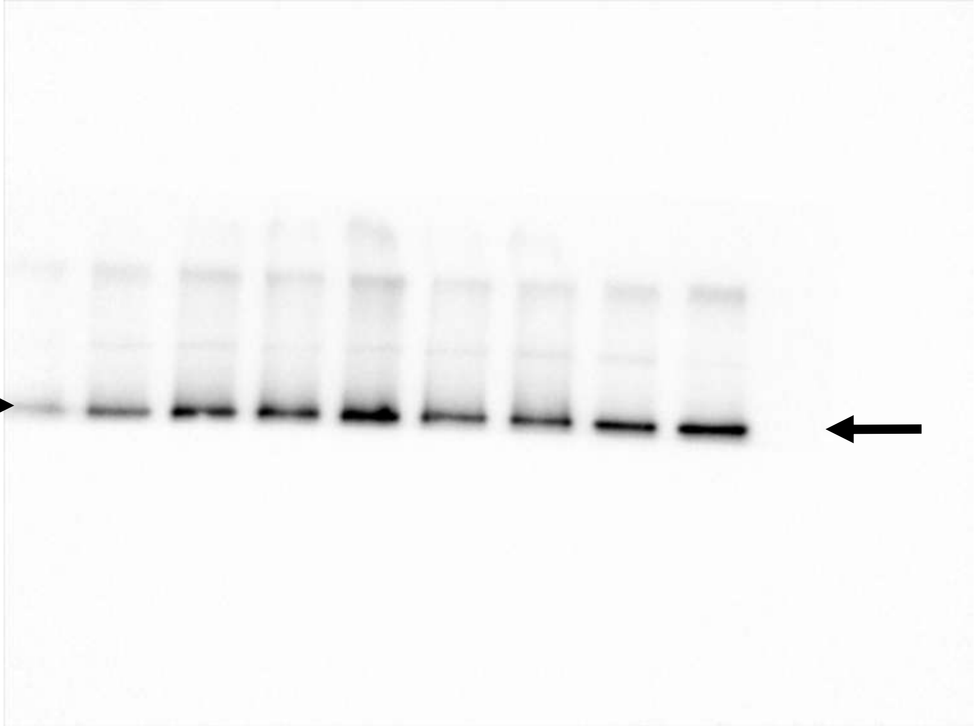

/Volumes/Seagate/Graduate Program/Research/SPECIFIC AIM 3/Western blotting/WB/Western blotting copy_2-5-18/Chemi Images/GA-1-31-18/RIP/Selected_Exposure_4.1sec.scn

Acquisition Information

Imager	ChemiDoc™ XRS+
Exposure Time (sec)	4.105 (Signal Accumulation)
Flat Field	Applied (Lens)
Serial Number	721BR12059
Software Version	5.2.1
Application	Chemi
Excitation Source	No Illumination
Emission Filter	No Filter
Binning	3x3

β-Actin

MDA-MB-231

Image Informa

SYA014 (μM)

0	2	5	10

Last 4 blots (right side) are not part work

β-Actin
42 kDa

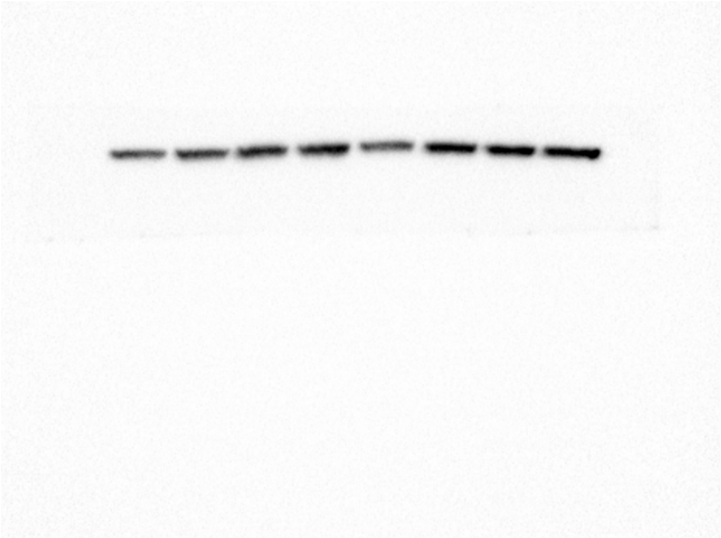

Figure 9 - G. Cell Cycle Proteins

P21

MDA-MB-231

SYA014 (μM)

0 2 5 10

Last 4 blots (right side) are not part of this work

Image Report: Pinaki 2018-03-17 14hr 31min_Exposure_56.9sec

P21
21 kDa

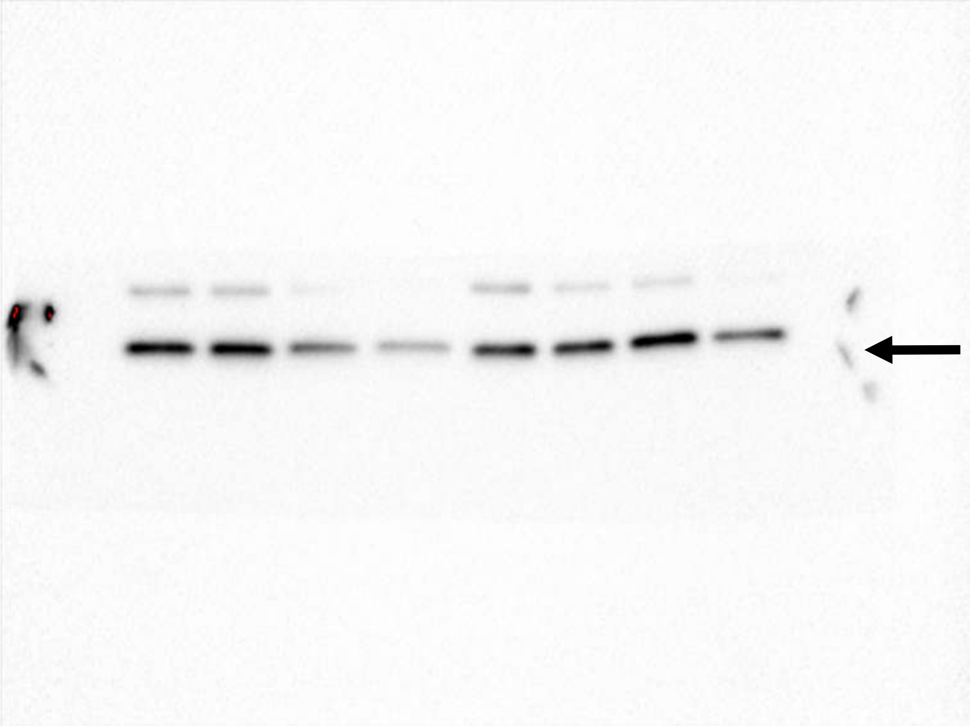

/Volumes/Seagate/Graduate Program/Research/SPECIFIC AIM 3/Western blotting/WB/Western blotting copy_2-5-18/Chemi Images/GA_3-7-18/GA_3-8-18/p-21 after Bcl-XL/Pinaki 2018-03-17 14hr 31min_Exposure_56.9sec.scn

Acquisition Information

Imager	ChemiDoc™ XRS+
Exposure Time (sec)	56.890 (Signal Accumulation)
Flat Field	Applied (Lens)
Serial Number	721BR12059
Software Version	5.2.1
Application	Chemi
Excitation Source	No Illumination
Emission Filter	No Filter
Binning	

P27

MDA-MB-231

SYA014 (µM)

0	2	5	10

Last 4 blots (right side) are [illegible]art of this

work

Image Report: Pinaki 2018-03-17 14hr 23min_Exposure_7.2sec

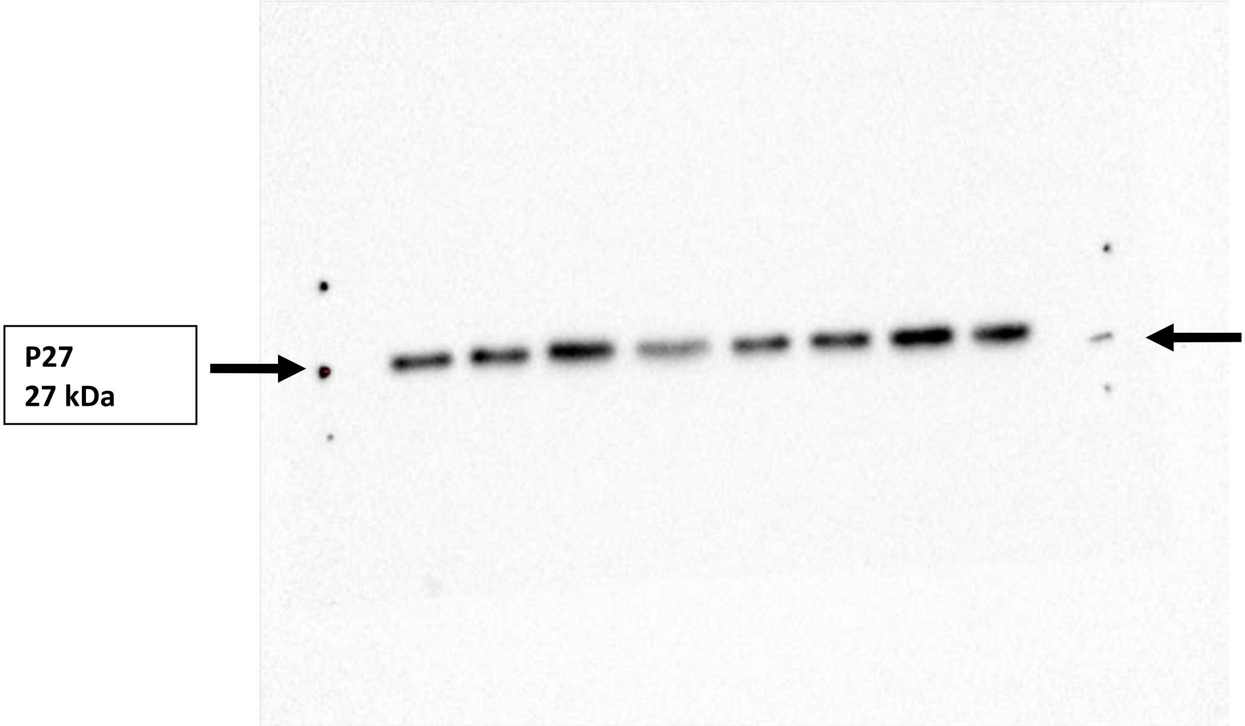

/Volumes/Seagate/Graduate Program/Research/SPECIFIC AIM 3/Western blotting/WB/Western blotting copy_2-5-18/Chemi Images/GA_3-7-18/GA_3-8-18/p-27 after Cylin D1/Pinaki 2018-03-17 14hr 23min_Exposure_7.2sec.scn

Acquisition Information

Imager	ChemiDoc™ XRS+
Exposure Time (sec)	7.210 (Signal Accumulation)
Flat Field	Applied (Lens)
Serial Number	721BR12059
Software Version	5.2.1
Application	Chemi
Excitation Source	No Illumination
Emission Filter	No Filter
Binning	3x3

CdK2

MDA-MB-231

SYA014 (μM)

0 2 5 10

Last 4 blots (right side) are not part of this work

Image Report: Pinaki 2018-03-01 12hr 53min_Exposure_224.2sec

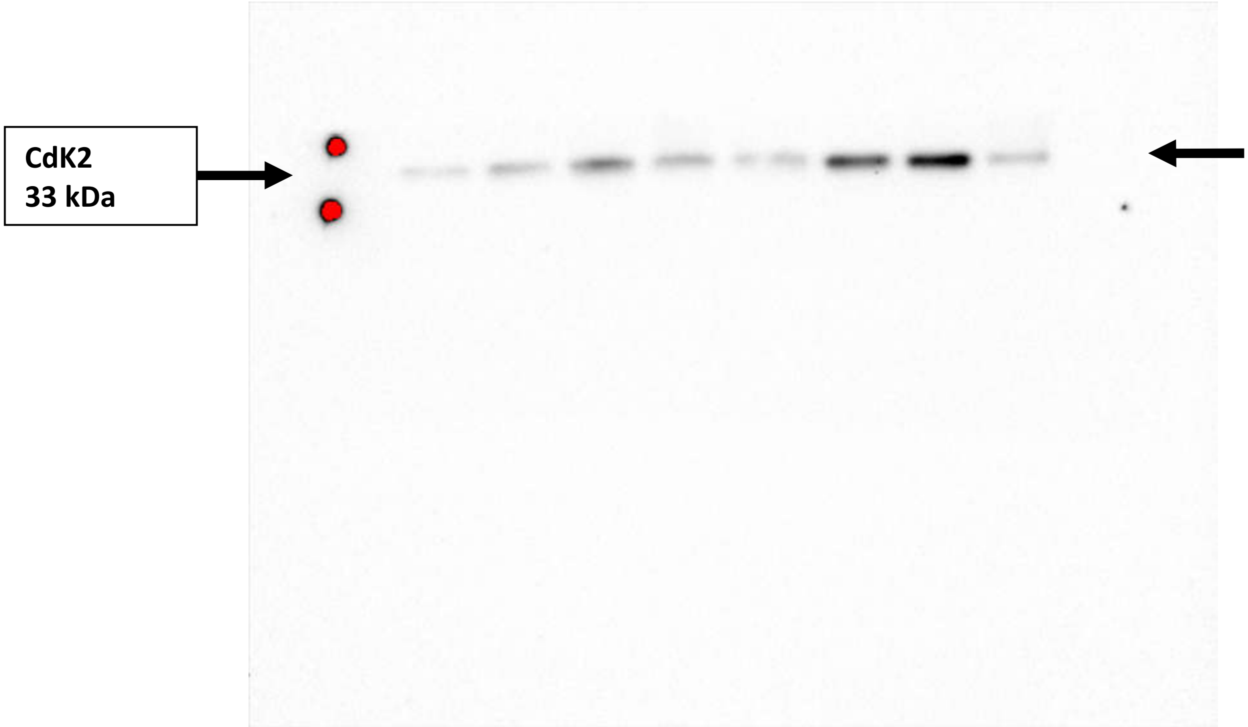

/Volumes/Seagate/Graduate Program/Research/SPECIFIC AIM 3/Western blotting/WB/Western blotting copy_2-5-18/Chemi Images/GA-2-20-18/CDK2/Pinaki 2018-03-01 12hr 53min_Exposure_224.2sec.scn

Acquisition Information

Imager	ChemiDoc™ XRS+
Exposure Time (sec)	224.203 (Signal Accumulation)
Flat Field	Applied (Lens)
Serial Number	721BR12059
Software Version	5.2.1
Application	Chemi
Excitation Source	No Illumination
Emission Filter	No Filter
Binning	

CdK4

MDA-MB-231

SYA014 (μM)

0 2 5 10

Last 4 blots (right side) are not part of this work

CdK4
30 kDa

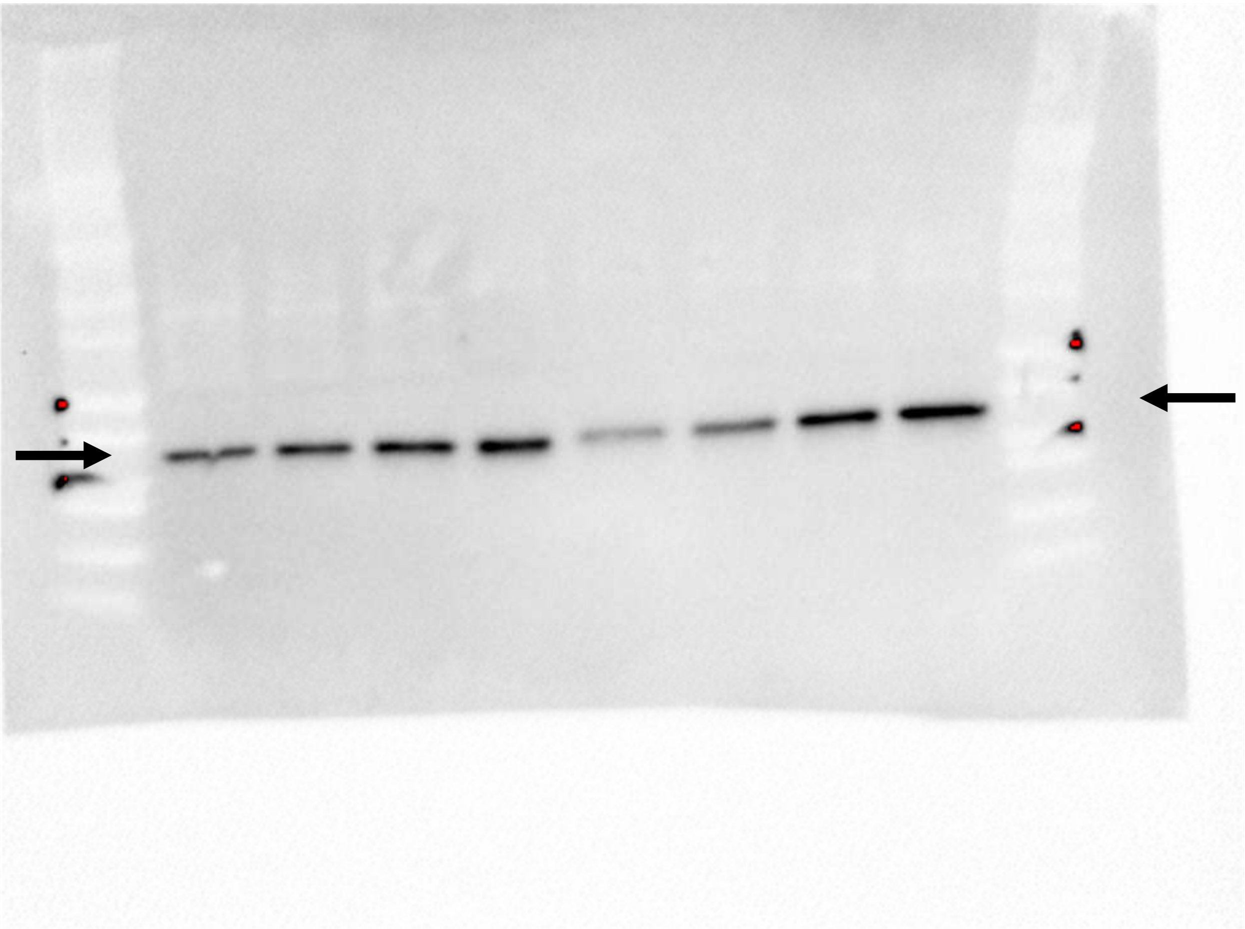

CyclinD1

MDA-MB-231

SYA014 (μM)

0 2 5 10

Last 4 blots (right side) are not part of this work

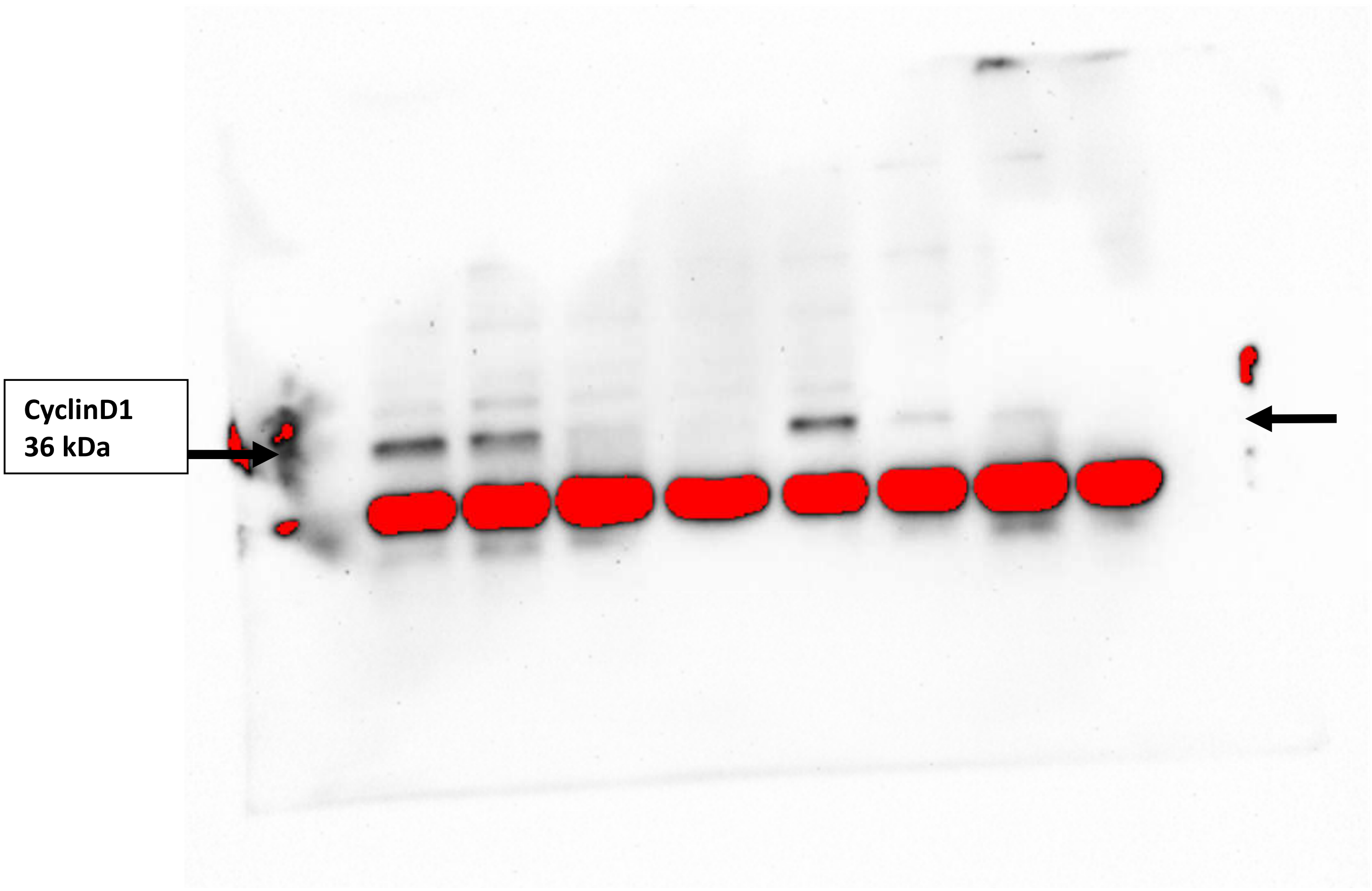

β-Actin

MDA-MB-231

SYA014 (μM)

0 2 5 10

Last 4 blots (right side) are not part of this work

β-Actin
42 kDa

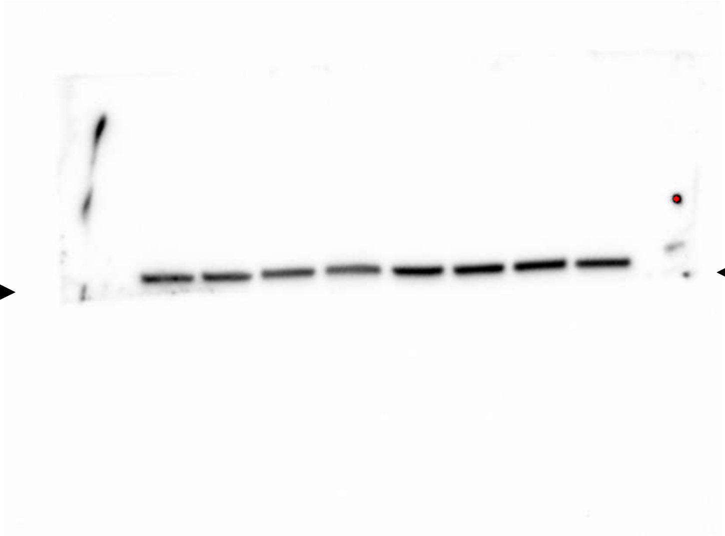

Supplement: Supplementary file 1 [file cancers-14-06047-s001.zip › cancers-2073976-supplementary.pdf]
